# Dr. J. W. Putnam

**Published:** 1908-03

**Authors:** 


### OBITUARY

Dr. J. W. Putnam, of Lyons, N. Y., died at the New York Central railway station on board a train passing through the city, February 9, 1908. The medical examiner was called and announced that the death was due to apoplexy. Dr. Putnam was sixty years old and had been a prominent physician in Lyons for many years.

